# Editorial: Advances in the Molecular Mechanisms in Gastrointestinal Tumorigenesis and Treatment

**DOI:** 10.3389/fonc.2021.827294

**Published:** 2022-01-05

**Authors:** Ruowen Zhang, Yu Zhang, Xiujuan Qu

**Affiliations:** ^1^ Research and Development Department, Jiahe Honsan (Shenzhen) Health Industry Group Co. Ltd., Shenzhen, China; ^2^ Institute of Biological Science, Jinzhou Medical University, Jinzhou, China; ^3^ Department of Medical Oncology, The First Affiliated Hospital of China Medical University, Shenyang, China

**Keywords:** gastrointestinal cancer, tumorigenesis, biomarker and diagnosis, therapeutic marker, mechanism study, immuno-therapy, tumor microenvironment, cancer metastasis

Gastrointestinal cancer (GIC) is a group of malignant tumors, including esophagus, stomach, liver, pancreas, as well as colorectal region. GIC accounts for more than 27% of the total newly diagnosed tumors worldwide, and 37% of tumor-related death events among all types of tumors. Totally, about 4.8 million new cases are yearly diagnosed and 3.4 million people lost their lives due to those malignancies every year (1). Moreover, the amount of either new or death cases are predicted to increase by 58% and 73% to 7.5 million and 5.6 million by 2040 due to the changes in the age composition and growth of the world population (2). In fact, the current poor prognosis and high mortality rate of GIC is mainly due to a high risk of metastasis, with its occurrence accompanied by lymphatic metastases and distant metastases (3–5). This serious pending burden forces us to re-consider how we deal with this challenge. In purpose of averting many future diagnoses and deaths, it is necessary to dedicate more work to the fundamental studies of these diseases, seeking reliable biomarkers benefiting to early diagnosis and preventative actions, and the development of innovative treatment strategies (6–9).

The Research Topic of this special issue takes an in-depth look at novel molecular mechanisms for gastrointestinal tumorigenesis and metastasis, as well as report the latest progress related to new biomarkers, therapeutic targets and development of new treatment strategies which will contribute to improved therapeutic efficacy and prognosis for GIC patients. A collection of original articles, systematic reviews, case reports and perspectives will provide readers with news on exciting breakthroughs in research into GICs including gastric cancer (GC), hepatocellular carcinoma (HCC), Cholangiocarcinoma (CCA), pancreatic ductal adenocarcinoma (PDAC), and colorectal cancer (CRC).

The majority of early stage of GIC metastases cannot be detected by computed tomography (CT), magnetic resonance imaging (MRI) or other traditionally morphological imaging approaches since the lesions are too small or they have not yet formed cancer nodules (10). However, there is still a rather limited number of useful biomarkers available that represents the risk of misdiagnosis leading to an ineffective therapeutic strategy and substantial problem for the management of GIC. Next generation sequencing (NGS) technology would appear to be a promising tool for GIC diagnosis. Chenming Wang et al. utilized on NGS RNA-based approaches to seek the significant regulators for liver metastases in colorectal cancer (CRC) patients. In their study, the elevated expression of Gankyrin and its positive correlation with disease progression as well as liver metastases in CRC patients were investigated. Gankyrin overexpression was shown to be an independent risk factor for the poor prognosis of CRC patients. Furthermore, CRC patients who did not have detectable liver metastases by conventional imaging examinations before their operation were followed up. It was discovered that patients with higher Gankyrin expression had a higher risk of liver metastasis and lower progression free survival (PFS) rates. Collectively, their data suggest that Gankyrin has the potential to serve as a biomarker for early detection of occult liver metastasis in CRC.

An ideal biomarker should be easily assayed with minimally invasive or non-invasive medical procedures but possess high sensitivity and specificity. Profiling of circulating RNAs, in particular long noncoding RNAs, have been used in a number of studies to identify novel and highly promising biomarkers for many pathologies including neurodegenerative diseases such as Alzheimer’s and Parkinson’s, brain injury and presence of tumors (11). Many of these molecules were determined to be associated with exosomes. Exosomes are extracellular vesicles with a diameter of approximately 30–100 nm (12). They can be secreted into the body fluid by many cells, including tumor and normal cells (13). Exosomes derived from cancer cells encapsulate various kinds of tumor-specific molecules, such as nucleic acid and proteins. Therefore, they can reflect biological characteristics of parental cells and interact with adjacent or distant cells to mediate information exchange (14). Miao Yu et al. presented the significant upregulation of exosomal FOXD2-AS1, NRIR, and XLOC_009459 in CRC, and revealed their potential as diagnostic biomarkers in clinic. Besides of detecting exosome, Haipeng Zhu et al. also reviewed the advanced clinical approaches in diagnosis of GICs at early stage including M2 Pyruvate Kinase (M2PK), Circulating Tumor Cell (CTC), Circulating Tumor DNA (CtDNA), microRNA (miRNA) and Circular RNA (circRNA). In fact, due to complex of the GICs, precise methodologies primarily depend on the personalized combination of M2PK, miRNAs, ctDNAs, cancer-produced exosomes, and CTCs with conventional tumor markers and serologically biochemical examinations that would be more beneficial in evaluation of treatment efficacy and prognosis.

Other problems for encountering the management of CIC, especially in PDAC and metastasis CRC (mCRC), are frequently adoptive chemotherapeutic resistance. Currently, over 50% CRC patients suffer the FOLFOX resistant that significantly compromised the therapeutic outcomes. It is reported that mechanistic rationale for acquired or inherent chemotherapeutic resistance to the anti-tumor effects of 5-fluorouracil (5-FU) that is linked to oncogenic GLI1 transcription activity and NBS1 overexpression. Patients with high levels of GLI1 also expressed high levels of NBS1. Non-canonical activation of GLI1 is driven through oncogenic pathways in CRC, like the BRAFV600E mutation. GLI1 was identified as a novel regulator of NBS1 and discovered that by knocking down GLI1 levels *in vitro*, diminished NBS1 expression, increased DNA damage/apoptosis, and re-sensitization of 5-FU resistant cancer to treatment was observed (15).

Cancer-associated gene (CAGE), a cancer/testis antigen, has been known to promote anticancer drug resistance for GIC therapy. However, the underlying mechanisms of CAGE-promoted anticancer drug resistance are still poorly understood. Minjeong Yeon et al. reported CAGE increases autophagic flux by binding to Beclin1 and inhibited the cleavage of PARP in response to anticancer drugs. MicroRNA array also revealed miR-181b-5p as a potential negative regulator of CAGE by binding to the promoter sequences. In addition, CAGE showed binding to the promoter sequences of phingosine 1-phosphate receptor 1 (SIPR1) which is a potential target for miR-181b-5p. Their study presents a novel role of the CAGE–miR-181b-5p–S1PR1 axis in anticancer drug resistance and autophagy.

Cisplatin is an important agent in first-line chemotherapy against gastric cancer (GC). However, consequential drug resistance limits its effectiveness for the treatment of GC. Mengyao Sun et al. presented the cisplatin resistant in gastric cancer is relative to up-regulation of exosomal levels of RPS3 protein. Their research focus on SGC7901R cell derived exosomes which were readily taken up by cisplatin sensitive SGC7901S cells, thus triggering off a phenotype of chemoresistance in the receptor cells. All their findings demonstrated that cisplatin resistant gastric cancer cells communicate with sensitive cells through the intercellular delivery of exosomal RPS3 and activation of the PI3K-Akt-cofilin-1 signaling pathway. Targeting exosomal RPS3 protein in cisplatin resistant gastric cancer cells may thus be a promising strategy to overcome cisplatin resistance in gastric cancer.

In addition to chemotherapeutic resistance, the uncontrollable invasion and metastasis are significant issues leading to the poor prognosis and high mortality rate. Follistatin-like 3 (FSTL3) is abundantly expressed in several solid tumors and participate in the regulation of cell metabolism. In our Research Topic, Yuqing Li et al. report that the expression level of FSTL3 in colon cancer specimens was significantly higher, compared to normal tissue and interestingly, the expression of FSTL3 was related to lymph node metastasis, tumor stage, tumor size and intravascular emboli (IVE). As an upstream molecular event, it was reported that transcriptional regulation of FSTL3 was highly dependent on YAP1 de-phosphorylation events and that increased FSTL3 expression readily activated the β-Catenin pathway, which is a well-known signaling hub that promotes EMT processes and aerobic glycolysis in cancer cells. FSTL3 expression strongly promotes migration, invasion and metastatic formation of CRC cells by directly activating β-Catenin -mediated EMT and aerobic glycolysis. Since the abundant FSTL3 expression is a poor prognostic factor, pharmacological targeting of YAP1 to inhibit FSTL3 expression is a promising therapeutic strategy to benefit CRC patients in clinic.

A pan-cancer analysis, which is also referred to as an analysis of molecular abnormalities among several cancer types, is able to identify the common features and heterogeneities of some vital dysregulated biological processes in diverse cancer cell lineages. The project of a pan-cancer analysis, including Cancer Cell Line Encyclopedia (CCLE) and The Cancer Genome Atlas (TCGA), has been established based on the different human cancer cell lines and tissues at epigenomic, genomic, proteomic, and transcriptomic levels. Typically, this method has been applied in more than 9,000 expression data of TCGA-derived cancer genes. Therefore, a pan-cancer analysis helps to illustrate the patterns that are beneficial for developing the combination treatments and individualized therapies. Recently, the development of immunotherapy using immune checkpoint inhibitors against programmed death 1 (PD-1) and its ligands, programmed death ligand 1 (PD-L1, or CD274) and PD-L2 (PDCD1LG2) has achieved breakthroughs in treating GICs. However, it remains largely unclear about the expression profiles of PD-1, CD274 and PDCD1LG2 in the context of human pan-cancer. Lin Dai et al. comprehensively analyzed the expression pattern of the PD-1 ligands and the clinical significance in the prognosis prediction among the seven types of gastrointestinal malignancies including cholangiocarcinoma (CHOL, N = 45), colon adenocarcinoma (COAD, N = 521), esophageal carcinoma (ESCA, N = 173), liver hepatocellular carcinoma (LIHC, N = 424), pancreatic adenocarcinoma (PAAD, N = 182), rectum adenocarcinoma (READ, N = 177), and stomach adenocarcinoma (STAD, N = 407) collected from The Cancer Genome Atlas (TCGA) and the Cancer Cell Line Encyclopedia (CCLE) database. The clear correlation has been figured out between CD274/PDCD1LG2 and the tumor infiltration level, tumor mutation burden (TMB), microsatellite instability (MSI), mismatch repair (MMR), and DNA methyltransferase (DNMT). Interestingly, CD274/PDCD1LG2 expression was highly correlated with TMB and MSI in colon adenocarcinoma. Since such the changes can be utilized by tumor cells to destroy the immunogenicity and the immune recognition mechanisms leading to the immune escape phenotypes, a combination with methylase inhibitor is expected to inject a new vitality into the cancer treatment. Their study helps to understand the vital roles of CD274/PDCD1LG2 in gastrointestinal tumor-immune interactions.

In conclusion, GICs are still severe burden today that continue to contribute 1.4–1.8 times as much to global cancer deaths as they do to cancer cases (1, 16). This is related to the fact that most esophageal, gastric, liver, and pancreatic cancers remain extremely difficult to detect early, and curative treatment options are either very limited or unavailable at the time of diagnosis. Therefore, we are responsible to contribute more work to handle this challenge. Hopefully, this Research Topic would benefit the GIC patients in preventing, diagnosing earlier and more accurately, and proposing new targeted and interdisciplinary therapeutic approaches.

## Author Contributions

All authors contributed to the article and approved the submitted version.

## Funding

YZ: Natural Science Foundation of China (31970840 and 91642117).

## Conflict of Interest

Author RZ was employed by Jiahe Honsan (Shenzhen) Health Industry Group Co. Ltd.

The remaining authors declare that the research was conducted in the absence of any commercial or financial relationships that could be construed as a potential conflict of interest.

## Publisher’s Note

All claims expressed in this article are solely those of the authors and do not necessarily represent those of their affiliated organizations, or those of the publisher, the editors and the reviewers. Any product that may be evaluated in this article, or claim that may be made by its manufacturer, is not guaranteed or endorsed by the publisher.
